# Dr. Kocher’s Visit to Albany

**Published:** 1907-08

**Authors:** 


### Dr. Kocher's Visit to Albany.

DR. Albert Kocher, the son of Prof. Theodor Kocher, the famous surgeon of Berne, Switzerland, visited the Albany Hospital June 17, 1907, as the guest of Dr. Albert Vander Veer and Dr. W. G. Macdonald. He demonstrated, before a number of the physicians of Albany, his father's method for the removal of the thyroid in a case of goitre, upon a patient of Dr. J. N. Vander Veer. Dr. Kocher, before operating, stated that the great experience of the Clinic at Berne was due to the general prevalence of goitre in Switzerland. He was convinced, however, that no country was free from goitre, as they had had patients from every country and since he had been in America he had seen many cases. They had operated upon over 3,000 patients in Berne and he himself had done 400 operations. The success of surgical procedure depended not alone upon the skill of the operator, but also upon the careful determination of the general physical condition of the patient as a guide to the extent of operative action.

The operation of Billroth differed from that of Kocher in that Billroth removed the capsule with the thyroid, whereas Kocher peeled out the thyroid gland from the capsule and left the capsule. This resulted in diminution of hemorrhage and in preventing injury to the parathyroid glands. In Vienna sixteen per cent. of the cases operated upon had tetany while in Berne only four in a thousand. The operation is usually done under a local anaesthetic and at Berne one per cent. solution of novo-cocaine with four or five drops of adrenalin in 150 grams of this solution is the anaesthetic employed. A long semi-circular incision is made and the muscles are separated in order to expose the gland. Below the muscles lies the deep fascia which is the capsule of the gland. Below the muscles lies the deep fascia which is the capsule of the gland. After the first incision practically no cutting is done and the blunt instruments are almost entirely used. Hemorrhage is prevented as much as possible by clamping every visible vessel.—*Albany Med. Annals.*

---

THE only royal doctor in Europe, according to *The Tribune,* is Duke Carl Theodore of Bavaria. Few German princes have had a more romantic career than Duke Carl Theodore. He recently completed, with his wife as his assistant, his five thousandth operation for cataract. Poor people flock to his hospital, where they are treated free, the duke asking payment only from those who can easily afford it. He it was who successfully treated the Kaiser when, eight years ago, he was temporarily blinded by a swinging rope when cruising on the Hohenzollern in the North Sea.

---

DR. E. J. GILRAY, medical superintendent of the Erie County Hospital, lately appeared before the supervisors' committee which has been considering reform, and supported the contentions of the Charity Organisation Society except the one recommending that the hospital be taken from control of the almshouse keeper. Dr. Gilray recommended establishment of a separate kitchen in the hospital for consumptives, with a large and more nourishing diet; a supply of blankets, clothing and equipment for outdoor treatment; the construction of six shacks for outdoor treatment, each shack to accommodate four patients; and finally, a fence around the hospitals to keep the patients in the hospital and out of the saloons across the street. Dr. Gilray said these improvements would cost about $10,000. The cost of the shacks is estimated at $700 each, or $4,200 for the six; $3,000 for clothing for 60 patients and $2,000 for a fence. Let us hope that the supervisors will make appropriation and plan for all these improvements.

